# Crystallized Pickering Emulsions from Plant Oil as a Local Alternative to Palm Oil

**DOI:** 10.3390/foods14010104

**Published:** 2025-01-02

**Authors:** Cyrill Husmann, Tamara Schmid, Chiara Waser, Ivo Kaelin, Lukas Hollenstein, Nadina Müller

**Affiliations:** 1 Institute of Food and Beverage Innovation, Zurich University of Applied Sciences, Einsiedlerstrasse 35, 8820 Wädenswil, Switzerland; cyrill.husmann@bluewin.ch (C.H.); tamaraschmid@bluewin.ch (T.S.); 2 Institute of Computational Life Sciences, Zurich University of Applied Sciences, Schloss 4, 8820 Wädenswil, Switzerland; chiara.waser@zhaw.ch (C.W.); ivo.kaelin@zhaw.ch (I.K.); lukas.hollenstein@zhaw.ch (L.H.)

**Keywords:** Pickering emulsion, crystallization, viscosity, stability

## Abstract

Palm and palm kernel oils are preferred ingredients in industrial food processing for baked goods and chocolate-based desserts due to their unique properties, such as their distinctive melting behaviors. However, ongoing concerns about the social and environmental sustainability of palm oil production, coupled with consumer demands for palm oil-free products, have prompted the industry to seek alternatives which avoid the use of other tropical or hydrogenated fats. This project investigated replacing palm oils with chemically unhardened Swiss sunflower or rapeseed oils. Target applications were cookies and chocolate fillings. These oils were physically modified through emulsification, stabilized with finely ground oil press cake particles and crystallized waxes. Findings indicated that the emulsification of the oils increased viscosity and that the addition of wax was beneficial for long-term stability; however, the extent of this effect depended on the combination of oil and wax types. Furthermore, wax pre-crystallization and low shear during crystallization significantly improved emulsion stability. Despite these improvements, the resulting emulsions did not achieve sufficient stability and exhibited lower viscosity than palm oil. Future experiments should explore higher wax concentrations (1% or more) and develop analytical methods to better understand the wax composition and its role in oleogel formation.

## 1. Introduction

Palm and palm kernel oils are preferred ingredients in industrial food processing due to their beneficial properties, such as unique melting behaviors. Palm oil is extracted from the pulp and palm kernel oil from the kernel of the fruit of the oil palm. Both contain high amounts of solids, making them semi-solid at room temperature [1]. Typical palm oil applications in the food sector include baked goods, fried products, ice cream, and pasta [2]. In contrast, palm kernel oil is often used in chocolate fillings due to its melting profile within a narrower and lower temperature range [3]. Switzerland imported 15,490 tons of palm (kernel) oil in 2022, solely for use in the food industry [4]. However, in response to growing concerns over the social and ecological sustainability of palm oil cultivation and processing, the industry is seeking alternatives that do not involve other tropical or hydrogenated sources.

Particle stabilization, known as Pickering stabilization, is achieved by particles that are partially wetted by both the oil and water phases, leading to slower but more stable interfacial stabilization than emulsifiers [5]. Several critical factors influence the effectiveness of particles in stabilizing emulsions through Pickering stabilization, including particle size, concentration, shape, interfacial film rheology, and wettability [6,7,8,9,10]. To achieve effective stabilization, the particles should ideally be about ten times smaller than the emulsion droplets [11].

Enhanced crystallization by adding waxes to sunflower or rapeseed oils has been explored as a technological approach to replacing palm oil, as waxes possess high melting points (>40 °C) and are potentially kneadable at room temperature, making them interesting additives for modifying oil properties. The combination of wax and oil with induced crystallization has already been successfully tested in dough applications [12,13,14,15]. Furthermore, recent research on the formation of oleogels has shown that waxes act as structuring agents, or ‘oleogelators’, in the formation of oleogels [16]. The quality of these oleogels, which depends on the density of the crystal network and the extent of hydrogen bonding, differs among wax types due to their crystal morphology and composition [17,18], resulting in varying levels of elasticity in the final oleogel. For food applications, the choice of waxes is limited, with candelilla, carnauba, rice bran, and sunflower waxes often being used [18,19,20]. Plant-based waxes, present on plant surfaces as protection against adverse weather conditions [21], have also been found to act at low concentrations of 1–4 wt% [22] and exhibit good crystallization properties in oil due to their high melting components, long molecular chains, and low polarity [23].

In food systems, two distinct crystallization processes are typically encountered: spontaneous melt crystallization and crystallization from a supersaturated solution. Crystallization from a supersaturated solution relies on the solubility characteristics of the solute within the solvent. When molten fat cools below the melting point of its highest melting triglyceride (TAG), a phenomenon known as undercooling occurs, resulting in the fat becoming supersaturated with the highest melting TAGs and triggering nucleation [24]. For melt crystallization, on the other hand, a less extensive under-cooling of the system below its melting temperature suffices, creating the necessary driving force for crystal formation [25].

To date, neither the use of native waxes from oil extraction press cakes nor the targeted crystallization of particle-stabilized emulsions has been described in the literature. Besides traditional Pickering stabilization using solid particles, stabilization using high-melting surfactants can be achieved through solidification directly at the oil–water interface, as emulsions cool after homogenization. The resulting crystals typically consist of mono- or multilayers, with thicknesses ranging from nanometers to low micrometer ranges. This crystallization process is applied in various oil-based food products, such as table spreads, where crystals adsorbed at the interface surround dispersed water droplets, significantly enhancing emulsion stability [26].

In this study, chemically unhardened Swiss organic sunflower or rapeseed oils, i.e., oils that did not undergo a hydrogenation process, were physically modified through emulsification, using finely ground particles from the oil press cake for stabilization, followed by controlled cooling and shearing to induce crystallization. The additional stabilization of the emulsions was achieved by optionally adding natural wax components present in the organic press cakes and oils before crystallization. The choice of raw materials, i.e., sunflower and rapeseed oils and their press cakes, was based on their high importance for Swiss agronomy and the edible oil industry. The aim of the described modifications of plant oils through emulsification and crystallization was an increase in viscosity and a maximized emulsion stability. The final end goal of the physical modification was to develop an oil that is modified through purely physical processing but is sufficiently stable and viscous to be considered as an alternative ingredient for palm fat in baked and sweet goods.

## 2. Materials and Methods

### 2.1. Raw Materials

#### 2.1.1. Oils

Rapeseed Oil: High oleic, low linoleic (HOLL) quality, conventional cultivation (Florin AG, Muttenz, Switzerland).

Sunflower Oil: Classic quality type “Knospe”, organic cultivation (Nutriswiss AG, Lyss, Switzerland).

#### 2.1.2. Press Cakes

Rapeseed Press Cake: Classic quality, conventional cultivation (Florin AG, Muttenz, Switzerland).

Sunflower Press Cake: Classic quality type “Knospe”, organic cultivation (Nutriswiss AG, Lyss, Switzerland).

#### 2.1.3. Waxes

Sunflower Wax: (Armonia GmbH, Sargans, Switzerland).

Rapeseed Wax: Rapeseed wax pastilles (Graine Creative, Preuilly-sur-claise, France).

Microcrystalline Wax: Type Sasol 3971 (Sasol Limited, Sandton, South Africa).

### 2.2. Production Methods

The emulsion preparation and the selection of an emulsion ratio of 15:85 W/O was guided by the experiments and results of Schmid et al. [27]. A wax concentration of 0.5%, was chosen, since preliminary trials indicated that this concentration yielded satisfactory crystallization results. A comprehensive investigation into the influence of varying wax concentrations on the crystallization process was beyond the scope of this study and is suggested for future research.

#### 2.2.1. Types of Crystallized Oils and Emulsions

**Figure 1:**
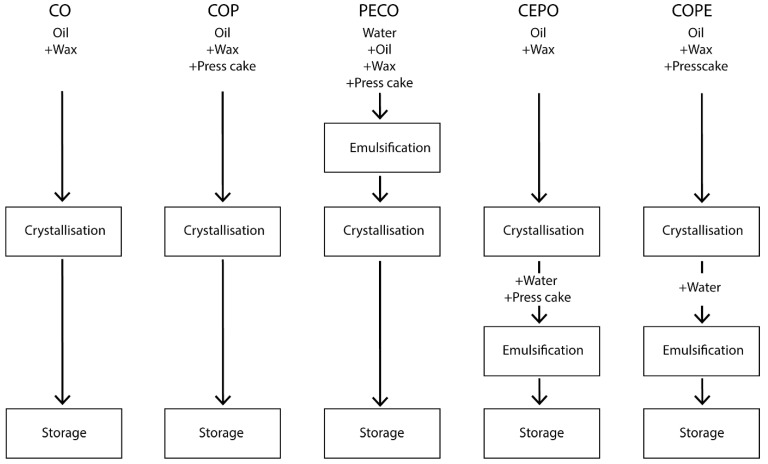
Summary of the different emulsification and crystallization process steps, where CO stands for ‘Crystallized Oil’, COP for ‘Crystallized Oil with Press Cake’, PECO for ‘Particle-Stabilized Emulsified Crystallized Oil’, CEPO for ‘Crystallized Emulsified Particle-Stabilized Oil’, and COPE for ‘Crystallized Oil with Particle Emulsification’.

In the following section, a short description of the different emulsification and crystallization processes is provided and summarized in Figure 1, followed by a detailed description of the processes and process parameters employed.

CO/COP: CO refers to ‘Crystallized Oil’, and COP to ‘Crystallized Oil with Press Cake’. Both involve a crystallization process of oil and wax, with COP additionally containing press cake.

PECO: Stands for ‘Particle-Stabilized Emulsified Crystallized Oil’. Unlike CO and COP, which contain no water and therefore are not emulsions, PECO is one of three types of crystallized emulsions produced during the trials. The process involves emulsification followed by crystallization.

CEPO/COPE: CEPO stands for ‘Crystallized Emulsified Particle-Stabilized Oil’, and COPE is ‘Crystallized Oil with Particle Emulsification’. CEPO is made from CO; the crystallized wax–oil suspension is first mixed with press cake, then water is added and emulsified to form a water-in-oil (W/O) emulsion. COPE is made from COP; the crystallized wax–press cake–oil suspension is mixed with water and emulsified at the same shear rate as CEPO.

#### 2.2.2. Preparation of Basic Suspension Containing Oil, Wax, and Optional Press Cake

For the basic suspension (oil phase), 99.5% (*v*/*v*) oil (sunflower oil or rapeseed oil) was measured and 0.5% (*w*/*v*) wax (rapeseed wax, sunflower wax, or microcrystalline wax) was added to one part of the oil (1000 mL corresponding to 1/5th of the total oil). This portion containing the wax was heated to 85 °C and held at that temperature until the wax was fully melted, as indicated by the disappearance of any haziness. If press cake was added, the remaining 4/5th of the oil was mixed with 5% (*w*/*v*) press cake based on the total quantity of the oil–wax–water mixture (rapeseed press cake or sunflower press cake) in a mixer (Rotor Lips AG, Uetendorf, Switzerland, GT 800) until it was homogeneously dispersed. Subsequently, the mixture was directly used for the emulsification process (see Section 2.2.3) or the crystallization process (see Section 2.2.4).

#### 2.2.3. Preparation of the W/O Emulsion

The emulsification process applied depended on the type of crystallized emulsion. In the case of PECO, emulsification was performed with a rotor–stator device. For COPE or CEPO, emulsification was carried out with a lab polytron. These two emulsification processes are described in the following two subsections:

Emulsification with rotor–stator: 85% oil–wax–press cake phase and 15% tap water were emulsified using a rotor–stator device (Megatron MT-FM 50, configuration: MTG 45 FFV/6 So, Kinematica AG, Malters, Switzerland) at a shear rate of 95,086 s^−1^. After emulsification, the emulsion was pumped directly into the scraped surface freezer (SWT-20-RH, Kinematica AG, Malters, Switzerland) for crystallization.

Emulsification with small polytron: A total of 9.75 mL of the crystallized oil–wax–press cake suspension was added to a 50 mL falcon tube, followed by 5.25 mL tap water. After a rest period of 60 s to allow the water to settle, the two phases were emulsified at 13,000 rpm using a polytron (PT 2500 E, Configuration: PT-DA 12/2EC-E157, Kinematica AG, Malters, Switzerland). With a rotor radius of 0.007 m and a gap between the rotor and the stator of 0.0005 m, the shear rates were estimated to be 9529 s^−1^. The emulsification process was initiated in the oil phase, and the device was slowly lowered into the water phase to form a W/O emulsion.

#### 2.2.4. Crystallization with Scraped Surface Freezer

The crystallization of the basic suspension or the emulsion was performed using a scraped surface freezer (SWT-20-RH, Kinematica AG, Malters, Switzerland). The suspension or emulsion was pumped into the freezing area, where scrapers removed the frozen product from the wall. The scraped surface freezer was cooled by an external cooling device (Unistat 430, Peter Huber Kältemaschinenbau SE, Offenburg, Germany) using ethanol as the cooling agent, with the cooling temperature set to −30 °C. The flow rate of the suspension or emulsion was set to 30 L/h, resulting in a holding time of 36 s. To investigate the influence of the scraper speed and shear rate on emulsion stability and viscosity, the scraper speed varied between 100 rpm, 300 rpm, and 600 rpm. With a scraper radius of 0.056 m and a gap between the scraper and the jacket surface of 0.002 m, the shear rates were estimated to be 147 s^−1^, 440 s^−1^, and 880 s^−1^, respectively.

### 2.3. Analysis Methods

#### 2.3.1. Viscosity

After 48 h of storage at 18 °C, the viscosity, η [mPa s], was measured by running a logarithmic shear ramp at 20 °C from 1 to 10 s^−1^ (measuring 10 points each for one second), from 20 to 100 s^−1^ (measuring 9 points each for one second), from 200 to 1000 s^−1^ (measuring 9 points each for one second), and from 2000 to 3000 s^−1^ (measuring 2 points each for one second).

For this measurement, 19 mL of the prepared emulsion was transferred into the sample cup. The viscosity was then analyzed using a coaxial cylinder measuring system (CC27- SN71788 64353, Anton Paar AG, Graz, Austria) with a rheometer (MCR 702, Anton Paar AG, Graz, Austria). Measurements were performed at a precision of 4–5 significant figures, according to the manufacturer.

To characterize the viscosity as a function of the shear rate, Sisko’s model [28] was used:

(1) $$\eta \left(\dot{\gamma }\right)=\eta_{\infty }+k{\dot{\gamma }}^{n-1}$$

The model considers that the viscosity approaches a value of $\eta_{\infty }$ [mPa s] at very high shear rates and not necessarily zero. The amplitude k [mPa s] and index n [-] characterize the shear thinning behavior before the plateau at $\eta_{\infty }$ is reached. A higher index n means less shear thinning, and a higher amplitude k means a higher overall viscosity.

The model parameters $\eta_{\infty }$, k, n were estimated through a least-squares fit to the experimental data for each combination of factors at each time of measurement, using the SciPy package (version 1.13.0) in Python (version 3.12.1).

#### 2.3.2. Emulsion Stability

Emulsion stability was determined by filling 7.5 mL of the prepared emulsions into 15 mL Falcontubes and storing them at 18 °C. The volumes of the cream layer (oil phase), emulsion layer, and serum layer (water phase) were measured relative to the initial volume after 0 h, 48 h, 168 h, 336 h, and 504 h of storage, according to the protocol described by [29]. Measurements were performed at a precision of 3 significant figures. The emulsion index EI [%] is defined by

(2) $$EI_{t}=100\cdot \frac{V_{t}}{V_{0}}$$

where $V_{0}$ is the total volume immediately after emulsion formation and $V_{t}$ is the volume without any phase separation after a storage times (*t*) of 48 h, 168 h, 336 h, and 504 h, respectively.

The short- and long-term emulsion stability, indicated by the emulsion index EI, were characterized using a logistic model:

(3) $$\frac{dEI}{dt}=\frac{K}{T}\;EI\;\left(1-\frac{EI}{L}\right)$$

The initial value for $EI\left(0\right)$ was defined as 100%. The parameter L [%] describes the long-term behavior of the emulsion index, $EI(t)\underset{t\to \infty }{\to }L$. The time it takes for the emulsion index to fall to 90% is parameterized by the decay time T [h], such that $EI\left(T\right)=90\%$. Finally, K is a dimensionless quantity given by L through $K=\ln\left(L-100\right)-\ln\left(L-90\right)-ln(0.9)$. The specific solution to the above differential equation is

(4) $$EI(t)=\frac{L}{1+\left(\frac{L}{100}-1\right)e^{-Kt/T}}$$

The model parameters T and L were estimated through a least-squares fit to the experimental data for each combination of factors using the SciPy package (version 1.13.0) in Python (version 3.12.1).

### 2.4. Experimental Design

First, different emulsification setups (order of emulsification and crystallization steps and the scraper speed of the scraped surface heat exchanger) were compared with respect to emulsion stability and viscosity for both rapeseed and sunflower based recipes.

Subsequently, the effect of adding natural waxes on emulsion stability and viscosity was observed using the optimal parameter identified in the first step.

In both experimental stages, each combination of factors (formulation) was replicated three times on different days, and each analysis was repeated three times, leading to a total of *n* = 9 results for each formulation and time of measurement.

The data obtained from the two experimental designs were analyzed using Kruskal–Wallis tests (α=0.05) to assess the equality of distributions among groups according to the independent factors, followed by a pairwise post hoc unpaired Wilcoxon test (α=0.05). Significant differences were indicated with different letters (compact letter display), where only results with no overlap in letters differ significantly from each other (e.g., ‘a’ and ‘b’ are significantly different, whereas ‘a’ and ‘ab’ are not significantly different). For calculations and visualizations, the following software packages were used: R (version 4.2.1) and RStudio (version 2023.09.0) with the Hmisc (version 5.1-2), spatstat (version 3.0-8), multcompView (version 0.1-10), and ggplot2 (version 3.5.0) packages.

The relationships between the viscosity and emulsion stability characterization parameters (models (1) and (4)) were visualized using scatter plots and kernel density estimation for each pair of variables. Pairwise scatterplots and probability densities were produced using the Seaborn package (version 0.13.2), which internally uses SciPy to compute a kernel density estimate using Gaussian kernels with the default bandwidth following Scott’s rule.

## 3. Results and Discussion

### 3.1. Effect of Order of Emulsification and Crystallization on Emulsion Stability and Viscosity

**Figure 2:**
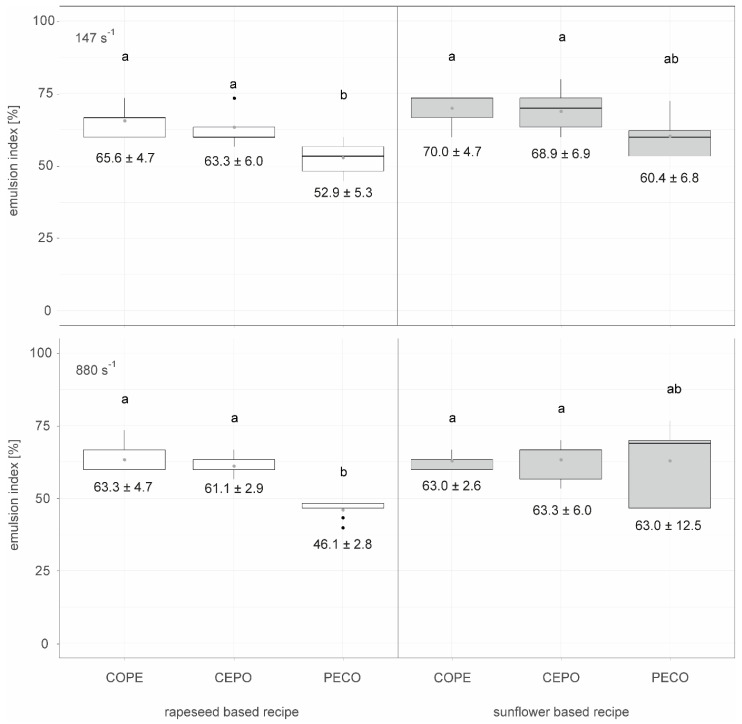
Emulsion stability indicated by the emulsion index after 504 h of storage at 18 °C for different types of emulsification processes (COPE: crystallized oil–wax–press cake suspension emulsified with water), CEPO: crystallized wax–oil suspension emulsified with water plus press cake) and PECO: particle stabilized W/O emulsions, additionally crystallized) crystallized at 147 s^−1^ and 880 s^−1^ in the surface scraped freezer, as well as differences between rapeseed oil and sunflower oil, n=9. Letters (a, ab, b) indicate groups of significance, i.e., group means not sharing any letter are significantly different by the Wilcoxon test with a *p*-value of 0.05.

Figure 2 shows the emulsion stability for different types of emulsification processes followed by crystallization at shear rates of 147 s^−1^ and 880 s^−1^ in the scraped surface freezer, as well as differences between rapeseed oil and sunflower oil, measured after 504 h (corresponding to three weeks) of storage at 18 °C (n=9).

At both shear rates, 147 s^−1^ and 880 s^−1^, both the rapeseed and the sunflower-based emulsions using the COPE and CEPO method had a significantly higher emulsion index after 504 h storage time than the rapeseed-based emulsion using the PECO method. The sunflower-based emulsion using the PECO method is not significantly more or less stable than the other emulsions.

The emulsions, which consist of crystallized oils and water, produced at a shear rate of 147 s^−1^ (boxplots in the upper row of Figure 2) were generally slightly more stable than the comparable emulsions produced at 880 s^−1^. This could be attributed to the fact that excessive shear can disrupt crystal structures, generate heat, and hinder further crystal growth, as well as impede the development of the fat’s macrostructure. In contrast, a low shear rate is beneficial for the nucleation process by enhancing heat and mass transfer, effectively reducing the formation of layers. Moreover, a low shear rate promotes faster crystal growth through several mechanisms, such as the alignment of triacylglyceride (TAG) molecules within the shear field and an increase in available growth sites due to crystal breakage [25].

As shown in Figure 2, the PECO method tends to produce less-stable emulsions compared to the CEPO and COPE methods, with a significant lower stability observed in rapeseed-based recipes. This could be due to the fact that, in the CEPO and COPE processes, the waxes were pre-crystallized before emulsification, whereas in the PECO process, the crystals formed directly during emulsification and cooling. Rousseau [26] describes different types of Pickering emulsions stabilized by fat crystals: one where fat crystals are formed during the cooling of the emulsion and another where fat crystals are formed before emulsion formation. While Rousseau did not observe significant effects from these emulsification settings on emulsion stability, Tenerio-Garcia et al. [29] achieved a stable W/O emulsion with gel-like properties that lasted 7 months by using pre-crystallized cocoa butter fat crystals in nanoplatelet form. These crystals stabilized the interface and formed an inter-droplet fat crystal network, effectively interlocking the water droplets.

Several critical factors influence the effectiveness of fat crystals in stabilizing emulsions through Pickering stabilization. These include particle size, concentration, shape, interfacial film rheology, and wettability [11]. This may explain the trend toward higher emulsion stability in sunflower-based recipes compared to rapeseed-based recipes, as the sunflower press cake had a significantly higher contact angle (90.58 ± 4.09°) than the rapeseed press cake (78.91 ± 3.51°) [27]. A contact angle close to or greater than 90° is favorable for stabilizing water-in-oil emulsion [27,30,31]. Moreover, Yilmaz [31] determined the gelling concentration of sunflower wax to be 1%, whereas that for rapeseed wax was 25% [32]. Therefore, the same amount of sunflower wax could lead to a higher viscosity of the emulsion and, consequently, slower coalescence of the droplets. A comparison with previous work on particle-stabilized emulsions using the same raw materials and emulsification processes shows a strong increase in the emulsion index for both raw materials. Specifically, an emulsion index of about 40% was retained after 168 h for emulsified rapeseed oil and press cake and of about 50% for sunflower oil and press cake [27].

**Figure 3:**
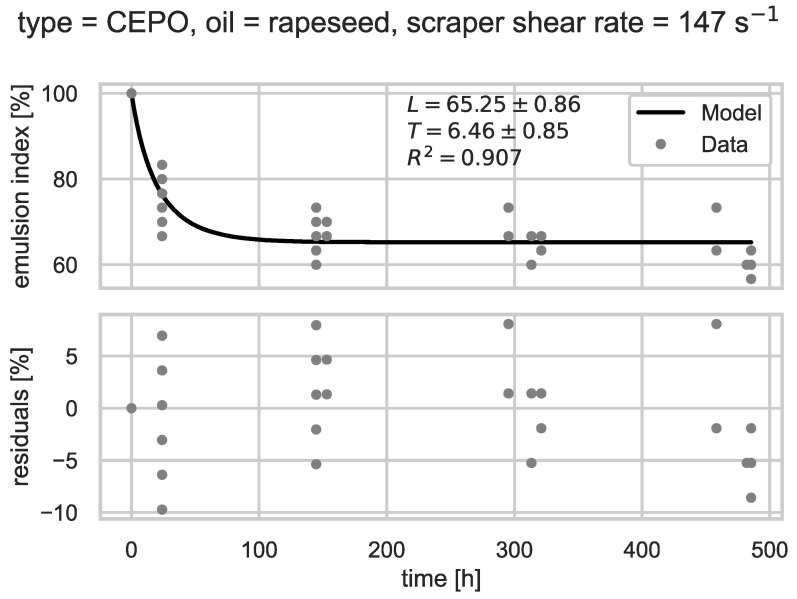
Emulsion index as a function of time for crystallization and subsequent mixing with press cake and emulsified (CEPO) rapeseed oil in a scraped surface freezer at a shear rate of 147 s^−1^. The datapoints are compared to an estimated logistic model. Long-term behavior L, decay time T until the emulsion index EI equals 90%, their 1σ-error, and the goodness of fit (R-squared) of the least-squares estimate are shown in the diagram.

The emulsion stability was further characterized using the logistic model described in Section 2.3.2, Equation (4), focusing on the long-term behavior parameter L and the decay time (T, the time it takes for the emulsion index to fall to 90%). Higher L- and T-values correspond to higher emulsion stability. The parameters L and T were estimated using a least-squares fit for each of the 18 factor combinations (process type, oil, shear rate). The resulting best-fit parameters, 1σ error estimates, and goodness of fit measures are listed in Table 1. The goodness of fit was quantified by the adjusted R-squared, with an average of $R^{2}=0.87$. An example of the decay of EI over time is shown in Figure 3.

**Figure 4:**
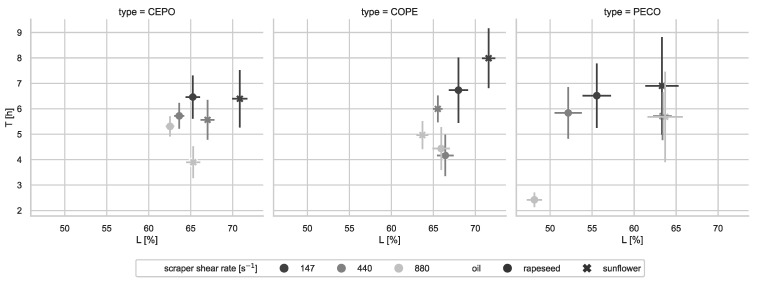
Relation between the emulsion stability (L-T-plane), process sequences (top CEPO, middle COPE, and bottom PECO), and shear rates [s^−1^] of the scraped surface heat exchanger (shades of gray) and oil recipe (rapeseed oil circles, sunflower oil crosses). The datapoints correspond to the least-squares estimate over 3 replications with 3 measurements each (n=9). The error bars show the 1σ estimation errors.

The T-L-scatterplot in Figure 4 illustrates the emulsion stability for the investigated process sequences (top: CEPO, middle: COPE, bottom: PECO), shear rates of the scraped surface heat exchanger (depicted by shades of gray), and recipes (rapeseed oil: circles; sunflower oil: crosses).

The long-term and short-term stability increased at lower shear rates. Stability was generally higher for the CEPO process compared to COPE and was significantly lower for PECO. Sunflower-based formulations showed better results (greater T and L values) at low shear rates compared to rapeseed-based formulations. For PECO, it was observed that crystallization led to the freezing of the water droplets, which melted during subsequent storage, affecting emulsion homogeneity. This was reflected in relatively large errors for the estimation of stability indicators T and L. The trends in the T-L-plane for PECO were comparable to those of the other two process types, CEPO and COPE.

**Figure 5:**
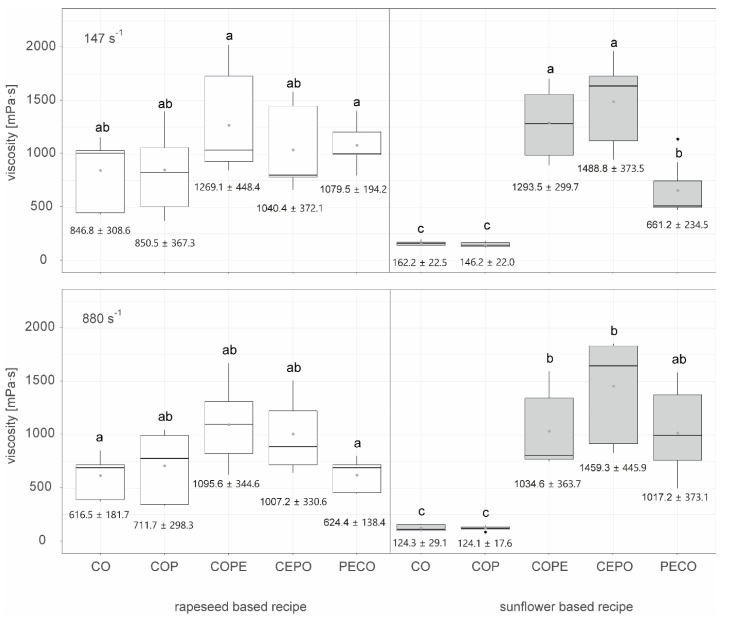
Viscosity measured after 48 h of storage at 18 °C for different types of crystallization processes (CO: crystallized oil, COP: crystallized oil with press cake) and emulsification processes (COPE: crystallized oil–wax–press cake suspension emulsified with water, CEPO: crystallized wax–oil suspension emulsified with water plus press cake, and PECO: particle stabilized W/O emulsions, additionally crystallized) crystallized at 147 s^−1^ and 880 s^−1^ in the surface scraped freezer, as well as differences between rapeseed oil and sunflower oil, n=9. Letters (a, b, c) indicate groups of significance, i.e., a group that does not share any letter is significantly different by the Wilcoxon test with a *p*-value of 0.05.

Figure 5 shows the viscosity measured at a shear rate of 1 s^−1^ for all the crystallized oils as well as emulsions 48 h after production at 147 s^−1^ (upper figure) and at 880 s^−1^ (lower figure) in the scraped surface freezer. Neither at 147 s^−1^ nor at 880 s^−1^ were significant differences in viscosity found for the rapeseed-based recipe.

For sunflower-based recipes, both at 147 s^−1^ and 880 s^−1^, CO and COP samples had significantly lower viscosities than the other crystallized oils/emulsions. At 147 s^−1^, the sunflower-based COPE and CEPO samples were significantly more viscous than the PECO samples.

As shown in Figure 5, a trend emerged where higher shear rates led to lower viscosities, likely due to the influence of shear on crystallization, which can alter the microstructure of fat and subsequently impact its physical and functional properties [25]. In contrast to the findings in this study, Werner-Cárcamo et al. [33] observed no effect of cooling and shear rate conditions on the polymorphic structure of the resulting oleogels [33].

There was a significant difference between the crystallized sunflower-based samples (CO and COP) and the crystallized and emulsified samples (COPE, CEPO, and PECO), with the latter group, having undergone an additional emulsification step, showing higher viscosity. A similar trend could be seen with the rapeseed-based samples, though the effect was less pronounced and not statistically significant. The increased viscosity in the additional emulsified samples (COPE, CEPO, and PECO) can be attributed to the addition of water and the emulsification process. The press cake particles have a high fiber content (sunflower press cake: 27.2 g/100 g; rapeseed press cake: 10.9 g/100 g), which swells upon contact with water. Moreover, Vidal et al. [30] reported a water holding capacity of 1.7 g/g for rapeseed press cake and 2.2 g/g for sunflower press cake [30]. The higher water holding capacity of sunflower press cake could be responsible for the slightly higher viscosity of the sunflower-based emulsions compared to the rapeseed-based ones.

Gu et al. [31] found that the addition of dispersed water to wax-based oleogels, compared to their non-emulsified counterparts, can hinder the growth of wax crystals, but the influence on resulting properties such as gelation ability and oil binding capacity depends on the type of structure formed by the respective waxes [30]. As a result of improved interfacial stabilization and a dense crystal network, wax-based emulsion gels exhibited solid-like properties and higher emulsion stability.

**Figure 6:**
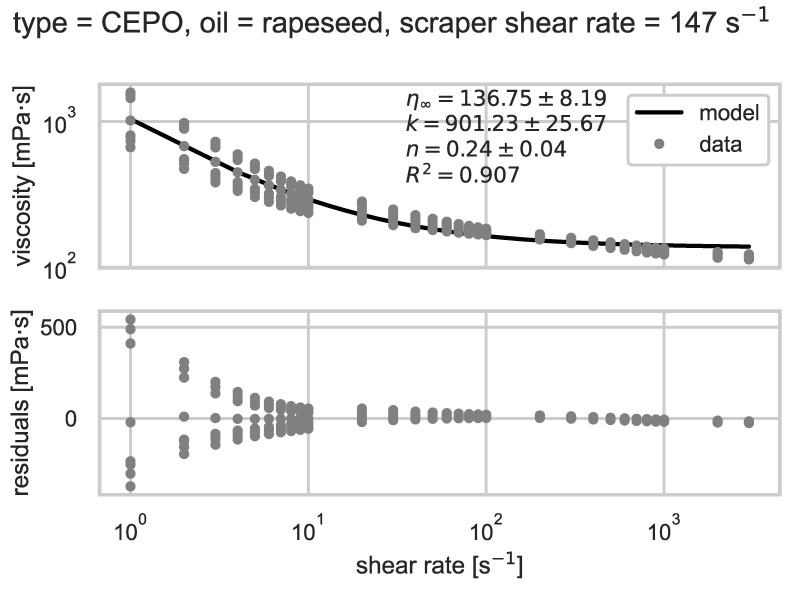
Viscosity as a function of shear rate for CEPO produced with the rapeseed oil shear rate of the scraped heat exchanger of 147 s^−1^). The data are compared to the estimated model for shear thinning viscosity. Corresponding model parameters, their 1σ-error, and the goodness of fit (R-squared) of the least-squares estimate are shown in the diagram.

The viscosity model described in Section 2.3.1, Equation (1) was estimated for all investigated formulations (see Table 2). Model accuracy was quantified by the adjusted R-squared, which averaged $R^{2}=0.88$. An example of viscosity dependence on shear rate is shown in Figure 6. In all experiments, the residuals indicate a small systematic deviation that the model cannot accurately reproduce. However, the model reproduces the overall behavior adequately for a basic characterization of shear thinning behavior using the parameters n, k, and $\eta_{\infty }$.

**Figure 7:**
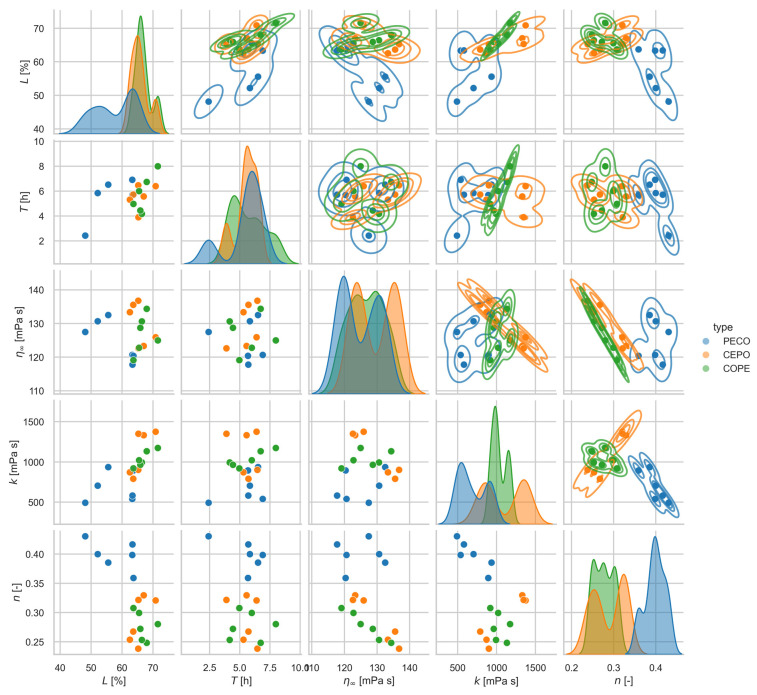
Pairwise scatterplot of the characterization of the emulsion stability (L and T) and viscosity (n, k, and$\;\eta_{\infty })$. The data were grouped by process type (shades of gray). The isolines of the estimated bivariate probability (kernel density estimates) are shown in the upper triangular matrix and the estimated univariate probabilities in the diagonal.

The scatter plots of all combinations of the characterization variables for emulsion stability (L and T) and viscosity (n, k and $\eta_{\infty }$), grouped by process type, are shown in Figure 7. The PECO process type shows substantially lower long-term stability L and a flatter viscosity curve (a higher index n, which means less shear thinning) than CEPO and COPE. As shown by the different directional correlation in the n-k-plane, the viscosity properties of PECO differ greatly from the other two processes. There is only a slight correlation between long-term emulsion stability (L and T) and the viscosity parameter k.

### 3.2. Effect of Wax Type on Emulsion Stability and Viscosity

**Figure 8:**
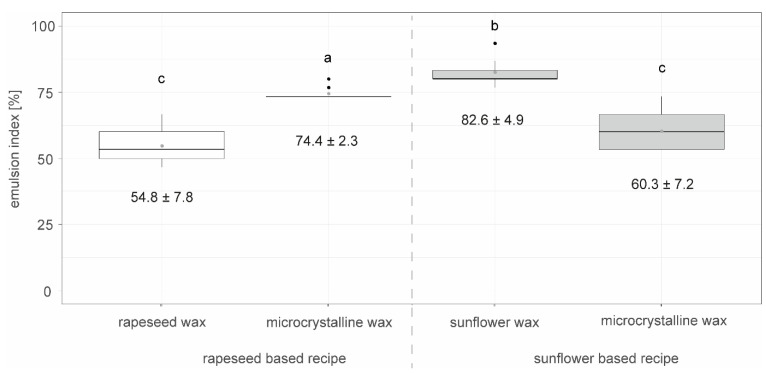
Emulsion stability indicated by the emulsion index after 504 h of storage at 18 °C for COPE: crystallized oil–wax–press cake suspension emulsified with water and crystallized at a shear rate of 147 s^−1^ in the surface scraped freezer containing rapeseed or sunflower oil and either 0.5% microcrystalline, sunflower, or rapeseed wax, n=9. Letters (a, b, c) indicate groups of significance, i.e., a group that does not share any letter is significantly different by the Wilcoxon test with a *p*-value of 0.05.

Figure 8 shows the emulsion stability of COPE emulsions crystallized at a shear rate of 147 s^−1^ in the scraped surface freezer, with different natural waxes, such as sunflower wax, rapeseed wax and microcrystalline wax, added at 0.5% and observed after 504 h of storage at 18 °C. The results for rapeseed oil combined with rapeseed wax and sunflower oil combined with sunflower wax differ from those in Figure 2 due to the use of a new batch of oil, highlighting natural variations in raw material quality. This variation underscores the need for the in-depth analysis of the detailed composition in future studies.

The sunflower-based recipe with sunflower wax had a significantly higher emulsion index after 504 h compared to the other emulsions. The rapeseed-based recipe with microcrystalline wax was significantly more stable than both the rapeseed-based recipe with rapeseed wax and the sunflower-based recipe with microcrystalline wax.

Building on previous studies by Schmid et al. [14], there was interest in comparing the effects of microcrystalline wax with natural waxes, such as rapeseed or sunflower wax [14]. Therefore, microcrystalline wax was used as a substitute for these natural waxes, while all other recipe components remained kept the same. For rapeseed-based formulations, the substitution of rapeseed wax for microcrystalline wax led to an increase in emulsion index while the opposite effect was found for the sunflower-based formulations. This suggests that the waxes interact differently with each oil. Notably, the sunflower wax-based emulsion was significantly more stable than all the others. A potential explanation for this might be the composition and the structure of the used wax types. From classic crystallized fat containing systems such as butter or chocolate, it is known that the microstructure of the solid phase and its interaction with the liquid phase affects the final product’s property and rheology [32]. Wax crystal shapes can vary from needle-like to platelet-like, dendritic, grain-like, or fiber-like, influencing their network forming ability and oil binding capacity [34,35]. Specifically, the surface area has been identified as a quantifiable attribute that enhances gelation capacity, with sunflower wax outperforming rice bran wax in this regard [36]. Pradhan et al. [37] compared the effects of candelilla wax, beeswax and rice bran wax on oleogelation, and found that the composition and structure of these waxes affected the required dosage and end-product characteristics across various applications, from baked goods to meat patties [37]. Much higher wax dosages were tested (3–7%) in a study by Hwang and Winkler-Moser [38] compared to the 0.5% doses used here, resulting in higher product firmness and melting temperatures than typical values for classic margarine. Typical compositional differences between wax types include variations in wax ester, hydrocarbon, and free fatty acid content [16]. Microscopic images (Supplementary Figure S1) show that upon melting wax in oil, microcrystalline wax formed separate needle-shaped crystals in both sunflower and rapeseed oil, while rapeseed wax in rapeseed oil was granular, and sunflower wax in sunflower oil formed dense network-like structures from fiber-shaped crystals.

**Figure 9:**
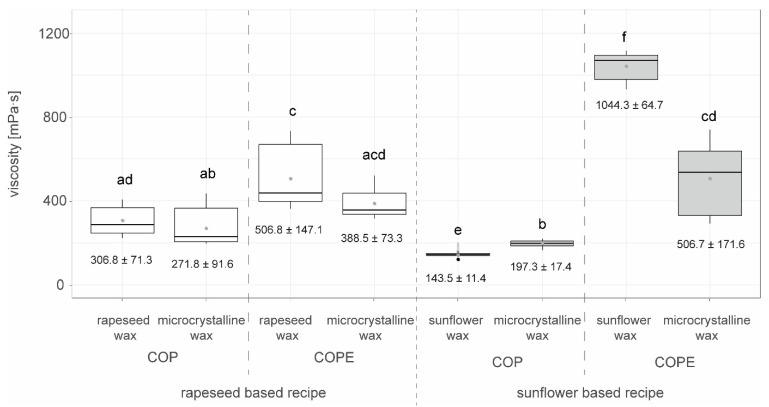
Viscosity measured after 48 h of storage at 18 °C for crystallization (COP: crystallized oil with press cake) and emulsification process (COPE: crystallized oil-wax-press cake-suspension emulsified with water), both crystallized at 147 s^−1^ in the surface scraped freezer for the raw materials rapeseed and sunflower oil and two types of wax, i.e., sunflower was and microcrystalline (MC) wax, added to each recipe, n=9. Letters (a, b, c, d, e, f) indicate groups of significance, i.e., a group that does not share any letter is significantly different by the Wilcoxon test with a *p*-value of 0.05.

The viscosity measured at a shear rate of 1 s^−1^ of all the crystallized oils and emulsions 48 h after production in the surface scraped freezer at a shear rate of 147 s^−1^ is shown in Figure 9 for different wax types.

The sunflower-based COPE with sunflower wax exhibited significantly higher viscosity than all other COP and COPE samples. The rapeseed-based COPE with rapeseed wax was significantly more viscous than the rapeseed-based COP with rapeseed wax, rapeseed-based COP with microcrystalline wax, sunflower-based COP with sunflower wax, and sunflower-based COP with microcrystalline wax, but was not more viscous than rapeseed-based COPE with microcrystalline wax or sunflower-based COPE with microcrystalline wax.

The COP samples generally had lower viscosity than the COPE samples (Figure 9), supporting the findings in Figure 5 where emulsified samples with crystallized wax components showed higher viscosity than samples that were only crystallized. This may again be due to the emulsification process and the addition of water, which leads to higher viscosity. Microcrystalline wax did not lead to higher viscosity compared to the natural waxes.

For oleogels comparable to the COPs, the type of wax and its composition and structure influence the end product quality. Few studies discuss the effect of different wax types on particle-stabilized emulsions comparable to the COPE type. Gu et al. [31] showed that wax crystals can play a dual role by stabilizing both the interface of water droplets and by forming a crystallized network, thus contributing to emulsion stability. Among the waxes tested, including sunflower, rice bran, carnauba, beeswax, candelilla, and sugarcane, all had plate-like structures except for the sugarcane, which had a floc-like structure, leading to a type of shell formation. A possible explanation for these differences might be the fibrous structure of the sunflower wax crystals [34], compared to the irregular shape of microcrystalline wax [39]. The fibrous structure of sunflower wax may contribute to a stronger network and higher viscosity. However, it remains unclear why a comparable effect was not found in non-emulsified crystallized oils (COPs) containing sunflower wax. No publications were found on the shape of rapeseed wax crystals, but our microscopic images (Supplementary Figure S1) show that rapeseed wax in rapeseed oil resulted in granular small crystals, from which no network formation can be expected. Further investigation into the shape of the wax crystals used and their network forming ability after emulsification is necessary to understand the different effects in COPs and COPEs in depth.

## 4. Conclusions

The results demonstrate that the emulsification of crystallized plant oils with particles from press cakes led to an increase in the viscosity of the products, likely due to the fibrous quality of the added particles and the structural differences between the pure crystallized oils and the corresponding emulsions. The addition of wax at a 0.5% dosage improved emulsion stability, with the extent depending on the combinations of oil and wax types. This suggests the need for a deeper understanding of the chemical composition of the waxes, including the waxy constituents of the press cakes, which is currently limited due to analytical constraints.

Furthermore, a lower shear rate during crystallization improved the stability of the crystallized oils and led to higher viscosity. It was also shown that pre-crystallization followed by emulsification resulted in higher emulsion stability than emulsification before crystallization. However, despite these improvements, complete emulsion stability was not achieved, and the viscosity did not reach levels comparable to palm oil.

For future trials, it is recommended to test higher dosages of waxes (1% or higher) to enhance their effect on viscosity and especially on emulsion stability to reach a storability of 4 weeks minimum and avoid the necessity for just-in-time production. Additionally, developing analytical methods for a detailed understanding of the composition and properties of the different wax components in the press cakes is crucial to comprehend their effect on the resulting oleogels.

## Figures and Tables

**Table 1:** Least-squares estimation results for the emulsion index stability model. Best-fit parameters, 1σ errors, and goodness of fit estimates for the emulsion index stability model for the 18 formulations investigated (3 repetitions with each 3 determinations at 4 points in time).

Type	Shear Rate [s^−1^]	Recipe	L [%]	$\sigma_{L}$ [%]	T [h]	$\sigma_{T}$ [h]	$R^{2}$
CEPO	147	rapeseed	65.2	0.860	6.46	0.848	0.907
CEPO	147	sunflower	70.9	0.906	6.39	1.13	0.858
CEPO	440	rapeseed	63.6	0.596	5.72	0.508	0.957
CEPO	440	sunflower	67.0	0.815	5.57	0.780	0.906
CEPO	880	rapeseed	62.5	0.508	5.31	0.396	0.970
CEPO	880	sunflower	65.3	0.822	3.90	0.626	0.913
COPE	147	rapeseed	68.0	1.14	6.73	1.29	0.823
COPE	147	sunflower	71.6	0.793	7.99	1.18	0.884
COPE	440	rapeseed	66.4	0.988	4.17	0.815	0.871
COPE	440	sunflower	65.5	0.557	6.00	0.526	0.958
COPE	880	rapeseed	65.9	1.03	4.44	0.841	0.865
COPE	880	sunflower	63.7	0.706	4.97	0.551	0.940
PECO	147	rapeseed	55.6	1.69	6.52	1.27	0.820
PECO	147	sunflower	63.3	1.97	6.90	1.92	0.681
PECO	440	rapeseed	52.2	1.62	5.84	1.01	0.855
PECO	440	sunflower	63.4	1.11	5.71	0.938	0.866
PECO	880	rapeseed	48.1	0.902	2.42	0.287	0.951
PECO	880	sunflower	63.7	2.09	5.68	1.78	0.642

**Table 2:** Least-squares estimation results for the viscosity model. Best-fit parameters, 1σ errors, and goodness of fit estimates for the 18 formulations investigated (3 repetitions with each 3 determinations at 4 points in time).

Type	Shear Rate [s^−1^]	Recipe	$\eta_{\infty }$ [mPa s]	$\sigma_{\eta_{\infty }}$ [mPa s]	k [mPa s]	$\sigma_{k}$ [mPa s]	n [-]	$\sigma_{n}$ [-]	$R^{2}$
CEPO	147	rapeseed	136.8	8.188	901.2	25.67	0.2383	0.03634	0.8442
CEPO	147	sunflower	125.9	10.04	1374	28.04	0.3208	0.02443	0.9131
CEPO	440	rapeseed	135.5	6.160	789.9	18.61	0.2675	0.02934	0.8879
CEPO	440	sunflower	123.3	8.417	1331	23.16	0.3296	0.02071	0.9352
CEPO	880	rapeseed	133.3	7.500	870.7	23.07	0.2536	0.03338	0.8623
CEPO	880	sunflower	122.6	12.08	1348	33.68	0.3216	0.02988	0.8753
COPE	147	rapeseed	134.3	9.786	1133	30.31	0.2482	0.03384	0.8601
COPE	147	sunflower	125.0	7.345	1174	21.80	0.2802	0.02291	0.9272
COPE	440	rapeseed	130.6	10.53	993.2	32.40	0.2532	0.04111	0.8052
COPE	440	sunflower	122.8	7.408	1021	21.39	0.2994	0.02547	0.9091
COPE	880	rapeseed	128.7	8.153	964.2	24.47	0.2721	0.03150	0.8722
COPE	880	sunflower	119.1	9.374	919.7	26.72	0.3077	0.03510	0.8386
PECO	147	rapeseed	132.5	5.314	935.5	13.17	0.3855	0.01618	0.9561
PECO	147	sunflower	120.6	6.920	538.6	16.68	0.3985	0.03535	0.8175
PECO	440	rapeseed	130.7	2.829	704.1	6.800	0.3999	0.01101	0.9788
PECO	440	sunflower	117.8	6.474	580.7	14.98	0.4166	0.02918	0.8651
PECO	880	rapeseed	127.5	4.928	491.4	11.03	0.4305	0.02522	0.8938
PECO	880	sunflower	120.4	10.61	893.9	27.70	0.3591	0.03616	0.8192

## Data Availability

The original contributions presented in the study are included in the article/Supplementary Material; further inquiries can be directed to the corresponding author.

## Supplementary Materials

The following supporting information can be downloaded at https://www.mdpi.com/article/10.3390/foods14010104/s1, Figure S1: Light microscope images of 0.5% wax melted in oil at 80 °C after cooling to room temperature for the combinations rapeseed wax in rapeseed oil (top left), microcrystalline wax in rapeseed oil (bottom left), sunflower wax in sunflower oil (top right), and microcrystalline wax in sunflower oil (bottom right).

## Abbreviations

CO	Crystallized Oil
COP	Crystallized Oil with Press cake
PECO	Particle-stabilized Emulsified Crystallized Oil
CEPO	Crystallized Emulsified Particle-stabilized Oil
COPE	Crystallized Oil with Particle Emulsified
