# Supplementary material for: Crystallized Pickering Emulsions from Plant Oil as a Local Alternative to Palm Oil
Source: Foods. 2025 Jan 2;14(1):104. doi: 10.3390/foods14010104 (PMC11719929; doi:10.3390/foods14010104)
Supplement: Supplementary file 1 [file foods-14-00104-s001.zip › foods-3355051-supplementary.pdf]

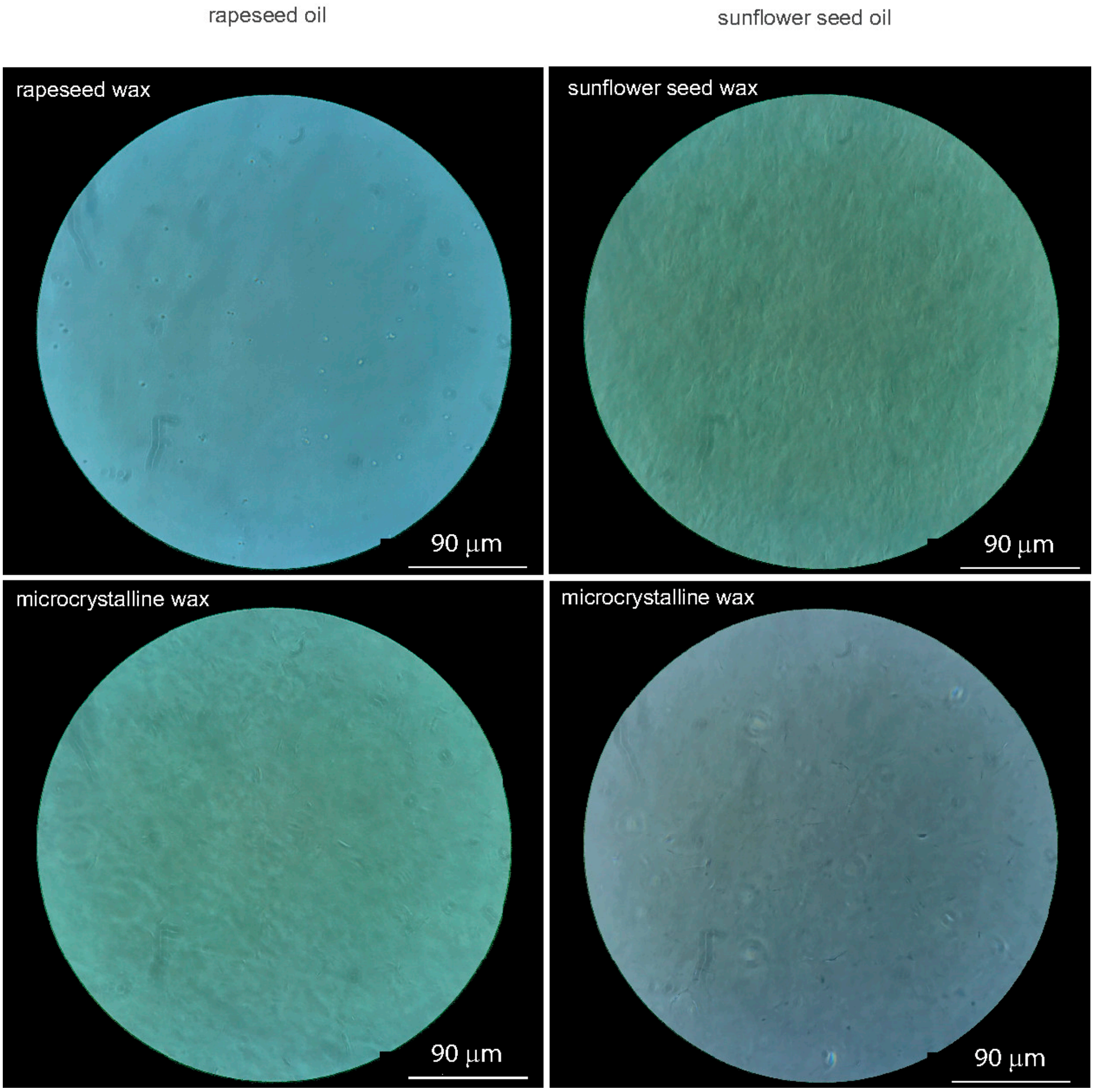

Supplementary Figure S1: Light microscope images of 0.5% wax melted in oil at 80 °C after cooling to room temperature for the combinations rapeseed wax in rapeseed oil (top left), microcrystalline wax in rapeseed oil (bottom left), sunflower wax in sunflower oil (top right), and microcrystalline wax in sunflower oil (bottom right).
